# Clinical significance of the expression of FOXP3 and TIGIT in Merkel cell carcinoma

**DOI:** 10.1038/s41598-023-40050-7

**Published:** 2023-08-12

**Authors:** Takeshi Iwasaki, Kazuhiko Hayashi, Michiko Matsushita, Daisuke Nonaka, Takamasa Matsumoto, Midori Taniguchi, Satoshi Kuwamoto, Yoshihisa Umekita, Yoshinao Oda

**Affiliations:** 1 Department of Anatomic Pathology, Graduate School of Medical Sciences, Kyushu University, 3-1-1, Maidashi, Higashi-Ku, Fukuoka, 812-8582 Japan; 2 Department of Pathology, School of Medicine, Faculty of Medicine, Tottori University, 86, Nishi machi, Yonago, Tottori 683-8503 Japan; 3 Department of Pathobiological Science and Technology, School of Health Science, Faculty of Medicine, Tottori University, 86, Nishi machi, Yonago, Tottori 683-8503 Japan; 4 Department of Cellular Pathology, The Guy’s and St. Thomas’ NHS Foundation Trust, London, UK

**Keywords:** Skin cancer, Tumour immunology

## Abstract

The pathogenesis of 80% of Merkel cell carcinoma (MCC) cases is associated with Merkel cell polyomavirus (MCPyV). Forkhead helix transcription factor P3 (FOXP3) and the T cell immunoreceptor with immunoglobulin and immunoreceptor tyrosine-based inhibition motif domains (TIGIT)–CD155 pathway, which are targets for immunotherapy, were assessed as prognostic factors of MCC. We analyzed mRNA expression data of 111 patients with MCC and performed immunohistochemical analysis to detect the expression of programmed death ligand 1 (PD-L1), CD8, FOXP3, TIGIT, and CD155 in 65 cases of MCC. In CD8 and FOXP3 immunostaining, the number of expressing-infiltrating cells was determined by dividing the region into tumor center and invasive front areas. FOXP3 expression was evaluated separately in cells with high and low intensities. Aberrant TIGIT expression and weak CD155 staining were observed in MCC cells. CD8- and FOXP3-positive cell infiltrations were higher in the invasive front than in the tumor center. Multivariate Cox hazard analysis revealed that high infiltration of cells with low-intensity FOXP3 expression in the invasive front is a favorable prognostic factor (p = 0.025). Thus, targeting TIGIT–CD155 signaling and FOXP3 as well as PD-L1 may be a therapeutic strategy for MCC.

## Introduction

Merkel cell carcinoma (MCC) is an aggressive neuroendocrine skin cancer. Merkel cell polyomavirus (MCPyV) is involved in the pathogenesis of about 80% of MCC cases^1^. The features of MCC differ depending on the MCPyV status in terms of morphology, prognosis, and molecular features and activation state of cancer signaling pathways that are potential therapeutic targets such as the phosphatidylinositol-3-kinase–Akt–mammalian target of rapamycin^2^, Janus kinase–signal transducers and activators of transcription^3^, mitogen-activated protein kinase–extracellular signal-regulated kinase^3^, and Notch^4^ signaling pathways. Recently, anti-programmed death receptor 1 (PD-1) or programmed death ligand 1 (PD-L1) monotherapy is considered standard therapy for unresectable, recurrent, or metastatic MCC, but primary resistance to immune checkpoint (IC) inhibitors remains a challenge.

A recent study reported that the immune status of the tumor microenvironment differs depending on the intratumor localization of various carcinomas, including esophageal squamous cell carcinoma^5^. In addition, we previously reported that MCC cases with low indoleamine 2,3-dioxygenase 1 expression in tumor cells and low tryptophan 2,3-dioxygenase 2 and aryl hydrocarbon receptor expression in tumor stroma are associated with good prognosis^6^. Based on the abovementioned reports, it is important to understand the intratumor heterogeneity of immune status and detailed immune status with and without MCPyV to improve the treatment of PD-1 and PD-L1 immunotherapy-resistant MCC.

The intratumoral presence of cluster of differentiation (CD) 8T cells does not necessarily mean that they can exert direct anti-tumor activities. Indeed, the function of intratumoral CD8 T cells is controlled by forkhead helix transcription factor P3 (FOXP3) regulatory T (Treg) cells and by immunosuppressive ligands such as PD-L1^7^.

FOXP3 is a master regulator in the development and control of Treg cells, which exert immunosuppressive functions and are related to the prognosis of various cancers including small cell lung cancer^8^, breast cancer^9^, and colon cancer^10^.

In addition, a previous study reported that FOXP3-positive T cells may function differently at different FOXP3 expression levels^10^. Further, it was reported that cells with low FOXP3 expression promote anti-tumor immunity, while cells with high FOXP3 expression inhibit anti-tumor immunity^10^.

T cell immunoreceptor with immunoglobulin and immunoreceptor tyrosine-based inhibition motif domains (TIGIT), which is the IC of T cells and natural killer (NK) cells, is often considered a marker of exhausted CD8-positive T cells, and it enables the maintenance of a quiescent state in CD8-positive T cells ^11^. However, expression of TIGIT is increased upon T cell activation, and it exhibits complex patterns in various T cell subsets. TIGIT mainly binds to CD155, interfering with T cell activation^12^. Thus, FOXP3 and TIGIT are considered attractive targets for cancer immunotherapy^13,14^.

The aim of this study was to evaluate the relationships between TIGIT, FOXP3, PD-L1, and MCPyV status and their values as potential therapeutic targets in MCC.

## Methods

### RNA expression profiling

We used a public ribonucleic acid sequencing dataset to evaluate mRNA expression in 111 MCC cases from the Finnish group study (Sequence Read Archive [SRA] accession number: PRJNA775071). The reads were mapped to the human reference genome (hg38) using STAR software^15^, and gene counting was performed using featureCounts^16^.

### Patient specimens

This study was approved by the institutional review boards of Kyushu University (study approval number: 2019–030) and Tottori University (study approval number: 1216). Informed consent for the research and publication of this study was obtained from all participants. Patient specimens (65 MCC cases) were prepared as formalin-fixed paraffin-embedded (FFPE) samples. Most of the samples were the same as those in our previous study^3^, and 21 samples were newly retrieved. We performed immunohistochemical staining of cytokeratin 20 and neuroendocrine markers such as synaptophysin, chromogranin A, and CD56, and the histological diagnoses were reconfirmed by four pathologists (TI, DN, SK, and KH). We also confirmed the negative status of thyroid transcription factor 1 to distinguish MCC from lung cancer metastasis. In a previous study, MCPyV infection status was analyzed using quantitative polymerase chain reaction, and immunostaining was performed using an antibody against MCPyV large T antigen (CM2B4). We used FFPE samples from 40 MCPyV-positive and 25 MCPyV-negative patients with MCC. Primary tumors were staged according to the latest American Joint Committee on Cancer (AJCC) staging system. The clinical background of the patients is summarized in Table 1.

**Table 1:** Comparison of clinicopathological parameters of Merkel cell carcinomas based on Merkel cell polyomavirus status.

Clinicopathological parameteres	MCPyV-negative	MCPyV-positive	p-value
Age (yo), mean ± SD	83.8 ± 8.9	78.3 ± 10.1	0.027*
Sex (male/female)	9/16	13/27	0.793
AJCC stage (I, II/III, IV)	20/5	32/6 [NA2]	0.741
Immunohistochemical results			
PD-L1 [TPS] (≥ 1%/ < 1%)	5/18 [NA 2]	4/34 [NA 2]	0.203
PD-L1 [CPS] (≥ 1%/ < 1%)	10/13 [NA 2]	21/17 [NA 2]	0.435
PD-L1 [IPS] (≥ 1%/ < 1%)	10/13 [NA 2]	20/18 [NA 2]	0.600
TIGIT (Tumor cells)	47.9 ± 7.0	50.2 ± 5.3	0.878
CD155 (Tumor cells) (+/−)	3/19 [NA3]	12/24 [NA 4]	0.128
Central tumor areas			
CD8 (Tcell)	304.3 ± 127.2	724.7 ± 87.2	0.002*
FOXP3 (High intensity)	22.9 ± 6.8	69.9 ± 13.4	0.099
FOXP3 (Low intensity)	67.3 ± 26.5	134.3 ± 14.8	0.004*
Invasive front areas			
CD8 (Tcell)	604.0 ± 150.9	896.3 ± 68.8	< 0.001*
FOXP3 (High intensity)	55.3 ± 16.3	69.9 ± 13.4	0.599
FOXP3 (Low intensity)	128.9 ± 27.9	134.3 ± 14.8	0.393

*TPS* tumor proportion score, *CPS* combined proportion score, *IPS* immune cell proportion score, *NA* not available.

*p < 0.05.

### Immunohistochemistry (IHC)

The primary antibodies used in this study are listed in Table S1. Immunostaining for FOXP3, TIGIT, CD8, and PD-L1 was performed as described in a recent study^17,18^. Immunostaining for PD-L1 expression was evaluated using tumor proportion score (TPS) and combined positive score (CPS). TPS was defined as the percentage of viable tumor cells with membranous staining, regardless of staining intensity. CPS was calculated by adding the number of PD-L1-expressing cells with membranous staining, including tumor cells, lymphocytes, and macrophages, and dividing the sum by the total number of viable tumor cells. The PD-L1-stained immune cells were evaluated using immune cell proportion score (IPS), which represents the estimated percentage (0%–100%) of immune checkpoints with membranous or cytoplasmic PD-L1 staining.

FoxP3-positive T cells and CD8-positive cells were evaluated in five different areas, including the parenchyma and stroma of the tumor center and the parenchyma and stroma of the invasive front. FoxP3-positive T cells were counted by differentiating between cells with high or low staining intensity.

CD8 staining was considered positive when the membranous staining result was positive. CD8-positive lymphocytes in tumors per five high-power fields were counted randomly, without including lymphoid aggregates. Tumor areas with artifacts and necrotic or apoptotic features were excluded.

The specimens were evaluated by three of the authors (TI, MM, and KH), and any differences in their evaluations were resolved by discussion between them to reach a consensus.

### Statistical analysis

The relationships between the mRNA expression of *CD274*, *FOXP3*, *CD8A*, *CD8B*, *TIGIT*, *PVR*, *CD28*, *ICOS*, *CTLA4*, and *PDCD1LG2* were analyzed using Spearman’s rank correlation coefficient. For clinicopathological analysis, Fisher’s exact test was used to analyze correlations between two dichotomous variables. Statistical analysis of IHC results of the resected specimens was performed using Wilcoxon test. Comparison of infiltrating cells in the invasive front and tumor center was performed using paired-sample Wilcoxon signed rank test.

The outcomes of IHC data were analyzed using linear regression analysis and Mann–Whitney U-test. Survival curves were constructed using Kaplan–Meier method. The CD8, CD155, and FOXP3 cut-offs were determined by plotting receiver operating characteristic (ROC) curves. Log-rank test was performed to analyze overall and disease-specific survival curves. The goodness of fit of each Cox model was evaluated using the likelihood ratio test, and the associations of individual variables with the study outcome were assessed using the forward Wald test. Covariates with p < 0.2 in univariate analysis were included in a multiple regression model. We used the JMP statistical software package (version 17; SAS Institute, Cary, NC, USA) for analysis, and p-values of < 0.05 were considered indicative of statistical significance.

### Ethics approval

This study was conducted in accordance with the principles of the Declaration of Helsinki, and the protocol was approved by the Ethics Committee of Kyushu University (No. 29-429 and 29-625).

## Results

### Relationship between mRNA expression of genes with immune checkpoints in MCC

**Figure 1:**
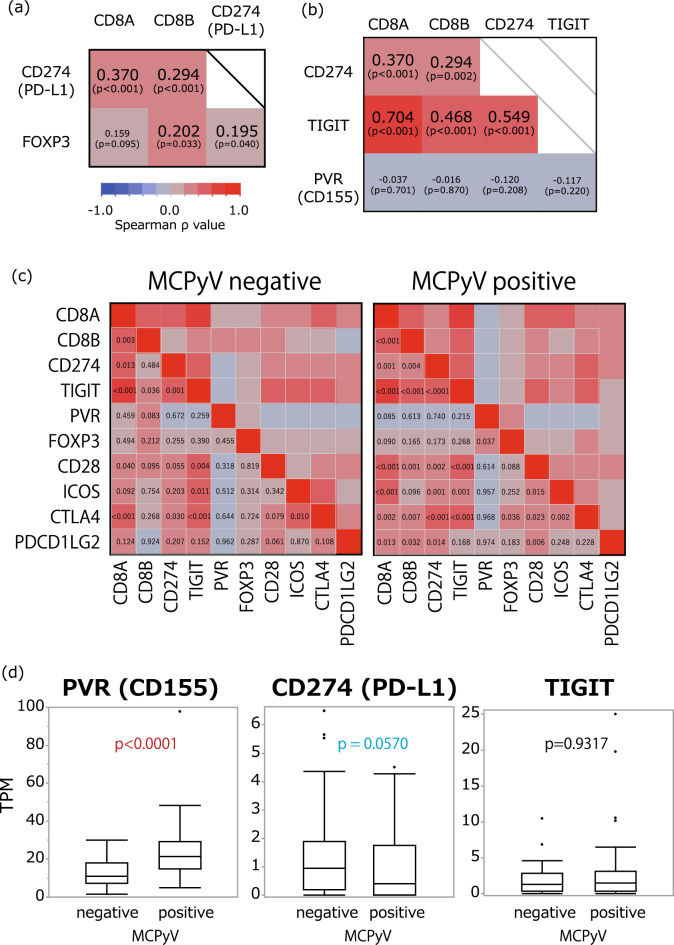
Relationships of the mRNA expression of genes. The figure shows the Pearson correlations between cluster of differentiation (*CD*) *274* (*PD-L1*) and *CD8A* and *CD8B* (**a**) and between *CD274* and *CD8A*, *CD8B*, T cell immunoreceptor with immunoglobulin and immunoreceptor tyrosine-based inhibition motif domains, and poliovirus receptor (*PVR*)(*CD155*) (**b**) and between, *CD8B*, *CD274*, *TIGIT*, *PVR*, *FOXP3*, *CD28*, *ICOS*, *CTLA4* and *PDCD1LG2* depend on MCPyV status (**c**). (**d**) Relationship between Merkel cell polyomavirus (MCPyV) status and the expression of *PVR*, *CD274* (*PD-L1*), and *TIGIT*. MCPyV-positive MCC cells have significantly higher mRNA expression of *PVR* and lower mRNA expression of *CD274* than MCPyV-negative MCC cells.

The analyzed data of mRNA expression obtained from the SRA are shown in Fig. 1. The values of Spearman (ρ) between *CD274, FOXP3, CD8A, CD8B, TIGIT, PVR, FOXP3, CD28, ICOS, CTLA4,* and *PDCD1LG2* are shown in Fig. 1a–c. The mRNA expression of *CD274* correlated positively and significantly with *CD8A , 8B* and *FOXP3* expression (Fig. 1a). The mRNA expression of TIGIT correlated positively with the expression of *CD8A, CD8B*, and *CD274* (Fig. 1b), regardless of the presence of MCPyV (Fig. 1c). The expression of PVR was not correlated with that of *CD8A, CD8B, CD274, TIGIT* (Fig. 1b), *CD28, ICOS, CTLA4,* and *PDCD1LG2*, regardless of the presence of MCPyV (Fig. 1c).

No correlation was observed between the expression of *TIGIT*, *CD28*, *ICOS*, *CTLA4*, and *PDCD1LG2* and MCPyV status (Fig. 1c). The expression of *PDCD1LG2* was positively correlated with that of *CD8A, CD8B, CD274*, and *CD28* in MCPyV-positive MCC, but not in MCPyV-negative MCC. Trends in gene expression correlations of other markers were generally independent of MCPyV status, except for *PDCD1LG2* expression (Fig. 1c). The relationships between MCPyV status and the expression of *PVR*, *CD274*, and *TIGIT* are shown in Fig. 1d. MCPyV-positive MCC cells expressed *PVR* significantly more than MCPyV-negative MCC cells (p < 0.0001) (Fig. 1d). MCPyV-negative MCC cells showed a higher expression of *FOXP3* than MCPyV-positive MCC cells (p = 0.0366). Further, MCPyV-negative MCC cells expressed *CD274* more than MCPyV-positive MCC cells, and the difference in *CD274* expression approached statistical significance (p = 0.057) (Fig. 1d).

### Clinicopathological data of MCC cases

The clinicopathological data of patients with MCC are summarized in Table 1. There were 43 female and 22 male patients, with a mean age of 80.5 years (median: 83 years; range: 46–100 years). The locations of the tumors are as follows: head and neck (35), trunk (2), extremity (28).

### Immunohistochemical findings

**Figure 2:**
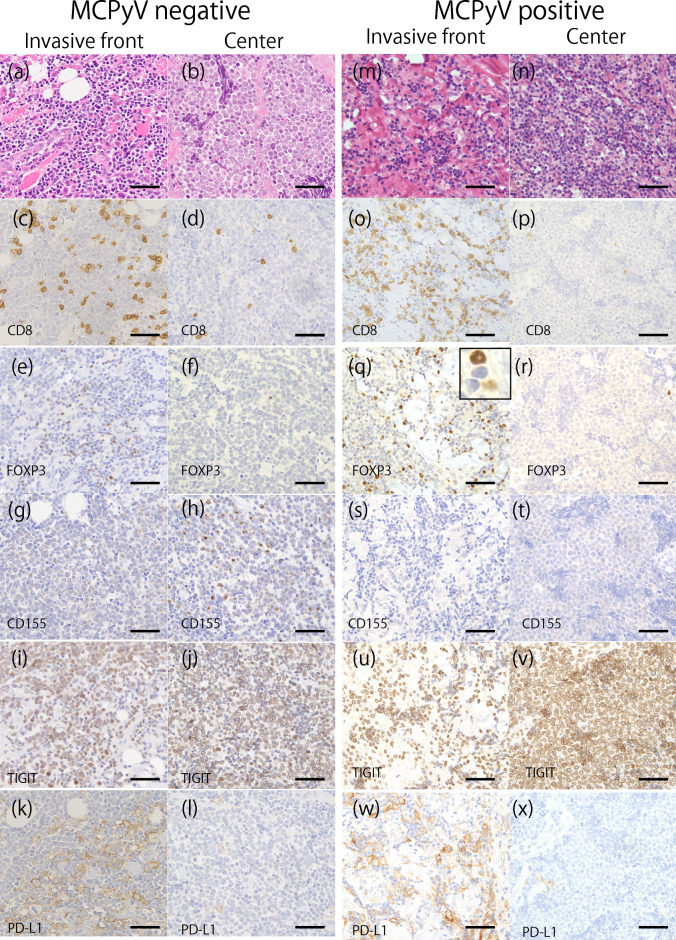
Representative immunostaining of MCPyV-positive and MCPyV-negative MCCs. MCPyV-negative MCC: (**a**–**l**). MCPyV-positive MCC: (**m**–**x**). Invasive front of tumor: (**a**,**c**,**e**,**g**,**i**,**k**,**m**,**o**,**q**,**s**,**u**,**w**). Tumor center: (**b**,**d**,**f**,**h**,**j**,**l**,**n**,**p**,**r**,**t**,**v**,**x**). (**a**,**b**,**m**,**n**) Hematoxylin and eosin staining of MCC. (c,d,o,p) Membranous staining of CD8 on lymphocytes. (**e**,**f**,**q**,**r**) Nuclear staining of forkhead helix transcription factor P3 (FOXP3) on lymphocytes. (**q**) (inset): Low- and high-intensity FOXP3 positive cells. (**g**,**h**,**s**,**t**) CD155 immunostaining showing cytoplasmic- and membranous-positive expression on tumor cells and a few lymphocytes. (**i**,**j**,**u**,**v**) TIGIT expression on tumor cells and immune cells. (**k**,**l**,**w**,**x**) Membranous staining of programmed death ligand 1 (PD-L1) on tumor cells and immune cells. The number of tumor-infiltrating CD8- and FOXP3-positive T cells was significantly higher in the tumor center than in the invasive front in both the MCPyV-negative (**c**–**f**) and MCPyV-positive (**o**–**r**) cases.

**Figure 3:**
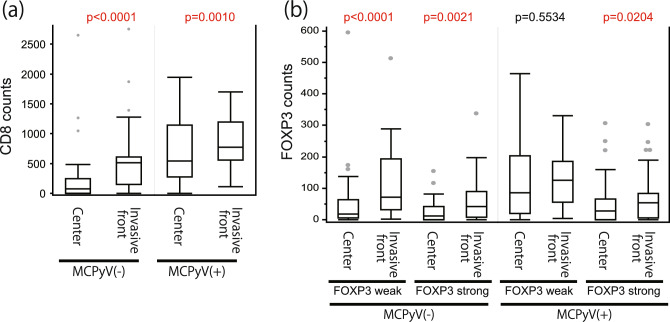
Summary of CD8 and FOXP3 immunohistochemical staining. The number of CD8-positive (**a**) and FOXP3-positive (high and low intensity) (**b**) cell infiltrates was plotted separately for the tumor center, invasive front, and MCPyV status. Paired-sample Wilcoxon signed rank test was used to evaluate statistical significance. The number of tumor-infiltrating CD8-positive T cells was significantly higher in the tumor center than in the invasive front in both MCPyV-positive (p = 0.001) and MCPyV-negative (p < 0.0001) cases (**a**). The number of FOXP3-positive T cells was significantly higher in the invasive front than in the tumor center in MCPyV-negative cases, for both low- (p < 0.0001) and high-intensity (p = 0.0021) FOXP3-expressing cells (**b**).

**Figure 4:**
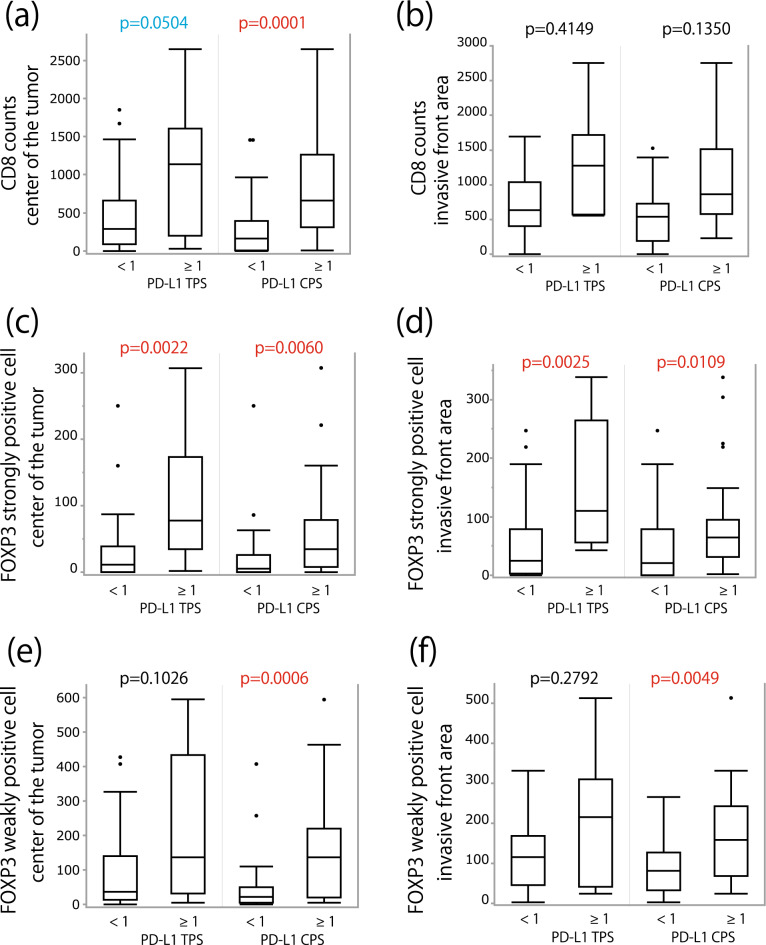
Relationship between PD-L1 expression and CD8 and FOXP3 immunohistochemical staining. The number of CD8-positive (**a**,**b**) and high-intensity (**c**,**d**) and low-intensity (**e**,**f**) FOXP3-positive cell infiltrates was plotted separately for the tumor center (**a**,**c**,**e**), invasive front (**b**,**d**,**f**), and PD-L1 expression. Paired-sample Wilcoxon signed rank test was used to evaluate statistical significance.

The representative results of IHC are shown in Fig. 2 and summarized in Table 1, Figs. 3, 4, and Supplemental Fig. 1. Membranous expression of PD-L1 was observed in tumor cells and inflammatory cells (Fig. 2k, l, w, x). PD-L1 immunostaining revealed nine (15%) TPS-positive (≥ 1%) cases, 31 (51%) CPS-positive (score ≥ 1) cases, and 30 (49%) IPS-positive (score ≥ 1) cases, with no correlation between MCPyV status and PD-L1 expression. Membranous CD8 expression (Fig. 2c, d, o, p) and cytoplasmic FOXP3 expression (Fig. 2e, f, q, r) were observed in the infiltrating inflammatory cells.

Further, the cytoplasm and the cytoplasmic membrane of tumor cells and a few lymphocytes showed weak CD155 staining (Fig. 2g, h, s, t). Aberrant TIGIT expression was observed in the cytoplasm of tumor cells (Fig. 2i, j, u, v).

### Relationship between MCPyV status and immunohistochemical results

The relationship between MCPyV status and immunohistochemical results is summarized in Table 1, Fig. 3, and Supplemental Fig. 1.

The number of tumor-infiltrating CD8-positive T cells was significantly higher in the invasive front than in the tumor center in both MCPyV-positive (p = 0.001) and -negative (p < 0.0001) MCC cases (Fig. 3a). In contrast, the number of FOXP3-positive cells was significantly higher in the invasive front than in the tumor center in MCPyV-negative cases, for both low- (p < 0.0001) and high-intensity (p = 0.0021) FOXP3-expressing cells (Fig. 3b). CD8-positive cell infiltration was significantly higher in MCPyV-positive MCC cases than in MCPyV-negative MCC cases in both the invasive front (p < 0.001) and the tumor center (p = 0.002) (Table 1). Infiltration of cells with low-intensity FOXP3 expression was significantly higher in MCPyV-positive MCC cases than in MCPyV-negative MCC cases in the central tumor area (p = 0.004) (Table 1). Similarly, infiltration of cells with high-intensity FOXP3 expression was higher in MCPyV-positive MCC cases than in MCPyV-negative MCC cases, but the difference was not statistically significant (p = 0.099) (Table 1). No correlation was observed between MCPyV status and infiltration of cells with FOXP3 expression in the invasive front. CD155 expression was higher in MCPyV-positive MCC cases than in MCPyV-negative MCC cases, and the difference approached statistical significance (p = 0.128, Table 1). However, there was no correlation between MCPyV status and TIGIT expression (Table 1).

### Relationships between PD-L1 expression status and CD8, FOXP3, CD155, and TIGIT expression revealed by IHC

The relationships between PD-L1 expression and CD8- or FOXP3-positive cell infiltrations are summarized in Fig. 4. The relationship between MCPyV status and the abovementioned IHC expression are shown in Supplemental Fig. 1. PD-L1 CPS-positive (≥ 1) cases showed significantly higher infiltration of cells with FOXP3 expression in the invasive front (Fig. 4d: high-intensity expression, p = 0.0109; Fig. 4f: low-intensity expression, p = 0.0049). In addition, the difference was statistically significant in MCPyV-negative cases, but not in MCPyV-positive cases (Supplemental Fig. 1d, f). CD8-positive cell infiltration in the tumor center was significantly higher in PD-L1 CPS-positive cases (Fig. 4a, p = 0.0001). The statistical difference was observed in both MCPyV-positive and -negative MCC cases (Supplemental Fig. 1a). TIGIT expression was also higher in PD-L1 CPS-positive cases than in PD-L1 CPS-negative cases, and the difference approached statistical significance (p = 0.135). PD-L1 was more frequently expressed in CD155-positive cases, but without statistical significance (PD-L1 TPS, p = 0.21; PD-L1 CPS, p = 0.23).

### Survival analysis

The areas under the ROC curves for survival analysis that show the cut-off values for CD8 and FOXP3 are presented in Supplemental Fig. 2. Based on the cut-offs of CD8 and FOXP3 in the tumor center and the invasive front, cases were divided into the high infiltrative group and the low infiltrative group (Fig. 4). Kaplan–Meier analysis and log-rank test (Supplemental Fig. 3) showed that low infiltration of CD8-positive cells and cells that weakly express (low-intensity) FOXP3 is a poor prognostic factor, regardless of the site of infiltration (tumor center: CD8, p = 0.0157 [e]; FOXP3, p = 0.0171 [d] and invasive front: CD8, p = 0.0149 [f] ; FOXP3, p = 0.0215 [b]). Low infiltration of cells that highly express (high-intensity) FOXP3 was found to be a significantly poor prognostic factor in the tumor center (p = 0.0458) (c). There is a trend toward poor prognosis in tumor-infiltrating areas, but without statistical significance (p = 0.1142) (a). Further, high TIGIT expression in tumor cells was found to be associated with favorable prognosis, but without statistical significance (p = 0.1680) (g). CD155 expression on tumor cells and PD-L1 expression on tumor cells or inflammatory cells or both did not correlate with prognosis (h–k). MCPyV-negative cases were significantly associated with unfavorable outcomes (p = 0.0145) (l).

A summary of the results of univariate and multivariate Cox regression analyses of the relationship between prognosis and the clinicopathological factors and IHC results is shown in Table 2. Multivariate analysis revealed that old age, advanced AJCC stage, and low infiltration of cells with low-intensity FOXP3 expression in the invasive front were found to be statistically significant unfavorable prognostic factors.

**Table 2:** Cox regression analyses of the relationships of disease specific survival with clinicopathological factors and immunohistochemical results.

Univariate	HR	P-value
Sex (male/female)	0.448	0.220
Age	1.087	0.014*
MCPyV (positive/negative)	0.024	0.016*
AJCC Stage (III, IV/I, II)	2.902	0.083
Immunohistochemical results		
PD-L1 [CPS] (≥ 1%/ < 1%)	1.416	0.560
PD-L1 [TPS] (≥ 1%/ < 1%)	0.479	0.481
PD-L1 [IC] (High/Low)	1.217	0.740
TIGIT (Tumor cells) (high/low)	3.278	0.196
CD155 (Tumor cells) (positive/negative)	1.391	0.600
Central tumor area		
CD8 (Tcell) (high/low)	0.242	0.025*
FOXP3 (Strong stained cell) (high/low)	0.289	0.044*
FOXP3 (Weak stained cell) (high/low)	0.181	0.013*
Invasive front area		
CD8 (Tcell) (High/Low)	0.235	0.040*
FOXP3 (Strong stained cell) (high/low)	0.301	0.102
FOXP3 (Weak stained cell) (high/low)	0.127	0.014*

Multivariate	HR	P-value
Age	1.162	0.047*
MCPyV (positive/negative)	0.473	0.845
AJCC Stage (III, IV/I, II)	47.263	0.016*
Immunohistochemical results		
TIGIT (Tumor cells) (high/low)	0.935	0.984
Central tumor area		
CD8 (Tcell) (high/low)	0.246	0.628
FOXP3 (Strong stained cell) (high/low)	4.73E-12	0.058
FOXP3 (Weak stained cell) (high/low)	8.49E-11	0.772
Invasive front area		
CD8 (Tcell) (High/Low)	2.729	0.461
FOXP3 (Strong stained cell) (high/low)	6.78E-10	0.238
FOXP3 (Weak stained cell) (high/low)	8.49E-11	0.025*

*p < 0.05.

## Discussion

In this study, we evaluated FOXP3 expression based on intensity in the tumor center and invasive front and found that infiltration of cells with weak (low-intensity) FOXP3 expression was significantly higher in MCPyV-positive MCC cases than in MCPyV-negative MCC cases.

Previous studies reported on the heterogeneous spatial distribution of tumor-infiltrating immune cells and their subtypes between the tumor centers and the invasive fronts of various tumors such as breast cancer^19^ and colorectal carcinoma^20^. The relationship between FOXP3 expression and the prognosis of MCC is controversial. Some studies reported that high intratumoral FOXP3-positive lymphocyte count was associated with favorable outcomes^21,22^, while other studies reported that FOXP3 expression did not correlate with prognosis^23,24^. T cells can be classified into three populations based on FOXP3 expression level and CD45RA expression^25^. The three populations are as follows: CD45RA-negative high-FOXP3-expression T cells (which are Treg cells), CD45RA-negative low-FOXP3-expression T cells (which are cytokine-secreting non-Treg cells)^25^, and CD45RA-positive low-FOXP3-expression T cells (which are resting Treg cells). A previous study on colorectal carcinoma reported that abundant infiltration of low-FOXP3-expression T cells was associated with significantly better prognosis than infiltration of high-FOXP3-expression Treg cells^10^. In other words, in terms of expression level, FOXP3-positive T cells have completely opposite effects on tumor prognosis, which is why FOXP3-positive T cells were analyzed separately by expression level in this study. Our study results showed that both high and low FOXP3-expressing cells correlated with favorable outcomes; moreover, infiltration of cells with high-intensity FOXP3 expression was associated with PD-L1 TPS positivity, whereas infiltration of cells with low-intensity FOXP3 expression was not associated with PD-L1 TPS. In addition, multivariate Cox regression analysis revealed that high infiltration of cells with low-intensity FOXP3 expression in the invasive front is a statistically significant favorable prognostic factor, whereas infiltration of cells with high-intensity FOXP3 expression in the invasive front has no correlation with prognosis. The above findings may indicate the functional differences in the expression level of FOXP3-positive cells in MCC.

The infiltration of high numbers of intratumoral CD8+ T cells into the MCC microenvironment has been reported to be associated with a favorable outcome^26^. We also confirmed the results via univariate Cox regression analysis. However, the association between CD8 infiltration in the invasive front and prognosis remains controversial. In our study, we showed that the high number of infiltrating cells in the invasive front of the tumor was related to a favorable outcome, but this finding was controversial^26^.

TIGIT is known to be an IC receptor present on activated T cells, Treg cells, and NK cells that negatively regulates T cell proliferation and its functions by downregulating T cell receptors and activating CD28 signaling. Since TIGIT binds with CD155 (PVR), competing with CD226 and interfering with the activation of T cells^12^, accumulation of TIGIT-positive T cells in tumors is associated with advanced disease, predicted early recurrence, and reduced survival rates in patients with colorectal cancer^27^. This study is the first to report high TIGIT expression on MCC cells. Only a few previous studies have reported on TIGIT expression on cells of tumors, such as melanoma^28^, undifferentiated pleomorphic sarcoma (UPS) ^18^, murine colorectal carcinoma^29^, and murine breast cancer cell lines^29^; however, the topic is not yet fully understood. Therefore, in this study, we focused on TIGIT expression on tumor cells. MCC cases with high TIGIT expression tended to have more favorable disease-specific survival than MCC cases with low TIGIT expression, but the difference was not statistically significant.

This study is also the first study to report CD155 expression on MCC cells. CD155 is highly expressed in various types of carcinomas and sarcomas, including pancreatic cancer, lung adenocarcinoma, and UPS, and is associated with tumor progression and unfavorable prognosis^18^. We showed that PVR (CD155 mRNA) expression is significantly higher in MCPyV-positive MCC cases than in MCPyV-negative MCC cases, and immunostaining showed the same trend, but the difference was not statistically significant. Further, CD155 expression was not found to be a prognostic factor.

Thus, immunostaining and RNA-seq data showed that the tumor microenvironment varies depending on the presence of MCPyV.

A limitation of this study was racial and regional differences: RNA-seq data were obtained from Finland, whereas immunostaining and prognostic analysis data were derived from Japan and the United Kingdom. We previously reported that MCPyV positivity in MCC was higher in patients from Japan than in those from the United Kingdom^3^. Thus, further integrated analysis of racial and regional differences is warranted.

Avelumab (a PD-L1 inhibitor) and pemrolizumab (a PD-1 inhibitor) were approved by the Food and Drug Administration for the treatment of metastatic MCC in 2017 and 2019, respectively. None of the patients in this study were treated with IC inhibitors. Our study findings confirmed that there is no correlation between PD-L1 expression and MCPyV status, which is consistent with the findings of a previous study^30^. Some studies reported that PD-L1 expression in MCC is associated with favorable outcomes^23^, but our data was not related to disease-specific survival.

In conclusion, high infiltration of cells with low-intensity FOXP3 expression in the invasive front is a favorable prognostic factor. Aberrant TIGIT expression is observed in MCC and may be associated with unfavorable outcomes. Targeting TIGIT–CD155 signaling and FOXP3 as well as PD-L1 may be a therapeutic strategy for MCC.

### Supplementary Information

Supplementary Information 1.

Supplementary Information 2.

Supplementary Information 3.

Supplementary Information 4.

## Data Availability

The datasets generated and/or analyzed during the current study are available from the corresponding author upon reasonable request.

## References

[1] Feng H, Shuda M, Chang Y, Moore PS (2008). Clonal integration of a polyomavirus in human Merkel cell carcinoma. Science.

[2] Iwasaki T (2015). Comparison of Akt/mTOR/4E-BP1 pathway signal activation and mutations of PIK3CA in Merkel cell polyomavirus-positive and Merkel cell polyomavirus-negative carcinomas. Hum. Pathol..

[3] Iwasaki T (2022). Merkel cell polyomavirus-negative Merkel cell carcinoma is associated with JAK-STAT and MEK-ERK pathway activation. Cancer Sci..

[4] Wardhani LO (2019). Expression of notch 3 and jagged 1 is associated with Merkel cell polyomavirus status and prognosis in Merkel cell carcinoma. Anticancer Res..

[5] Hatogai K (2020). Relationship between the immune microenvironment of different locations in a primary tumour and clinical outcomes of oesophageal squamous cell carcinoma. Br. J. Cancer.

[6] Wardhani LO (2019). Expression of the IDO1/TDO2-AhR pathway in tumor cells or the tumor microenvironment is associated with Merkel cell polyomavirus status and prognosis in Merkel cell carcinoma. Hum. Pathol..

[7] Apetoh L (2015). Consensus nomenclature for CD8(+) T cell phenotypes in cancer. Oncoimmunology.

[8] Bonanno L (2018). The role of immune microenvironment in small-cell lung cancer: Distribution of PD-L1 expression and prognostic role of FOXP3-positive tumour infiltrating lymphocytes. Eur. J. Cancer.

[9] Merlo A (2009). FOXP3 expression and overall survival in breast cancer. J. Clin. Oncol..

[10] Saito T (2016). Two FOXP3(+)CD4(+) T cell subpopulations distinctly control the prognosis of colorectal cancers. Nat. Med..

[11] Banta KL (2022). Mechanistic convergence of the TIGIT and PD-1 inhibitory pathways necessitates co-blockade to optimize anti-tumor CD8(+) T cell responses. Immunity.

[12] Anderson AC, Joller N, Kuchroo VK (2016). Lag-3, Tim-3, and TIGIT: Co-inhibitory receptors with specialized functions in immune regulation. Immunity.

[13] Blake SJ, Dougall WC, Miles JJ, Teng MW, Smyth MJ (2016). Molecular pathways: Targeting CD96 and TIGIT for cancer immunotherapy. Clin. Cancer Res..

[14] Manieri NA, Chiang EY, Grogan JL (2017). TIGIT: A key inhibitor of the cancer immunity cycle. Trends Immunol..

[15] Dobin A (2013). STAR: Ultrafast universal RNA-seq aligner. Bioinformatics.

[16] Liao Y, Smyth GK, Shi W (2014). featureCounts: An efficient general purpose program for assigning sequence reads to genomic features. Bioinformatics.

[17] Kawatoko S (2022). Solid-type poorly differentiated adenocarcinoma of the stomach: A characteristic morphology reveals a distinctive immunoregulatory tumor microenvironment. Pathol. Res. Pract..

[18] Ishihara S (2022). Clinical significance of signal regulatory protein alpha and T cell immunoreceptor with immunoglobulin and immunoreceptor tyrosine-based inhibition motif domain expression in undifferentiated pleomorphic sarcoma. J. Cancer Res. Clin. Oncol..

[19] Mani NL (2016). Quantitative assessment of the spatial heterogeneity of tumor-infiltrating lymphocytes in breast cancer. Breast Cancer Res..

[20] Jakubowska K, Koda M, Grudzinska M, Lomperta K, Famulski W (2021). Tumor-infiltrating lymphocytes in tissue material combined with systemic lymphocyte inflammation in patients with colorectal cancer. Mol. Clin. Oncol..

[21] Donizy P (2021). Prognostic role of tumoral PD-L1 and IDO1 expression, and intratumoral CD8+ and FoxP3+ lymphocyte infiltrates in 132 primary cutaneous Merkel cell carcinomas. Int. J. Mol. Sci..

[22] Sihto H (2012). Tumor infiltrating immune cells and outcome of Merkel cell carcinoma: a population-based study. Clin. Cancer Res..

[23] Guenole M (2021). The prognostic significance of PD-L1 expression on tumor and immune cells in Merkel cell carcinoma. J. Cancer Res. Clin. Oncol..

[24] Ricci C (2020). Prognostic impact of MCPyV and TIL subtyping in Merkel cell carcinoma: Evidence from a large European cohort of 95 patients. Endocr. Pathol..

[25] Miyara M (2009). Functional delineation and differentiation dynamics of human CD4+ T cells expressing the FoxP3 transcription factor. Immunity.

[26] Paulson KG (2014). CD8+ lymphocyte intratumoral infiltration as a stage-independent predictor of Merkel cell carcinoma survival: A population-based study. Am. J. Clin. Pathol..

[27] Liang R (2021). TIGIT promotes CD8(+)T cells exhaustion and predicts poor prognosis of colorectal cancer. Cancer Immunol. Immunother..

[28] Kawashima S (2021). TIGIT/CD155 axis mediates resistance to immunotherapy in patients with melanoma with the inflamed tumor microenvironment. J. Immunother. Cancer.

[29] Zhou XM (2018). Intrinsic expression of immune checkpoint molecule TIGIT could help tumor growth in vivo by suppressing the function of NK and CD8(+) T cells. Front. Immunol..

[30] Feldmeyer L (2016). Density, distribution, and composition of immune infiltrates correlate with survival in Merkel cell carcinoma. Clin. Cancer Res..

