# Supplementary material for: Clinical significance of the expression of FOXP3 and TIGIT in Merkel cell carcinoma
Source: Sci Rep. 2023 Aug 12;13:13114. doi: 10.1038/s41598-023-40050-7 (PMC10423247; doi:10.1038/s41598-023-40050-7)
Supplement: Supplementary file 2 — Supplementary Information 2. [file 41598_2023_40050_MOESM2_ESM.pdf]

Supplemental Figure 1

Relationship between PD-L1 expression and CD8 and FOXP3 immunohistochemical staining and MCPyV

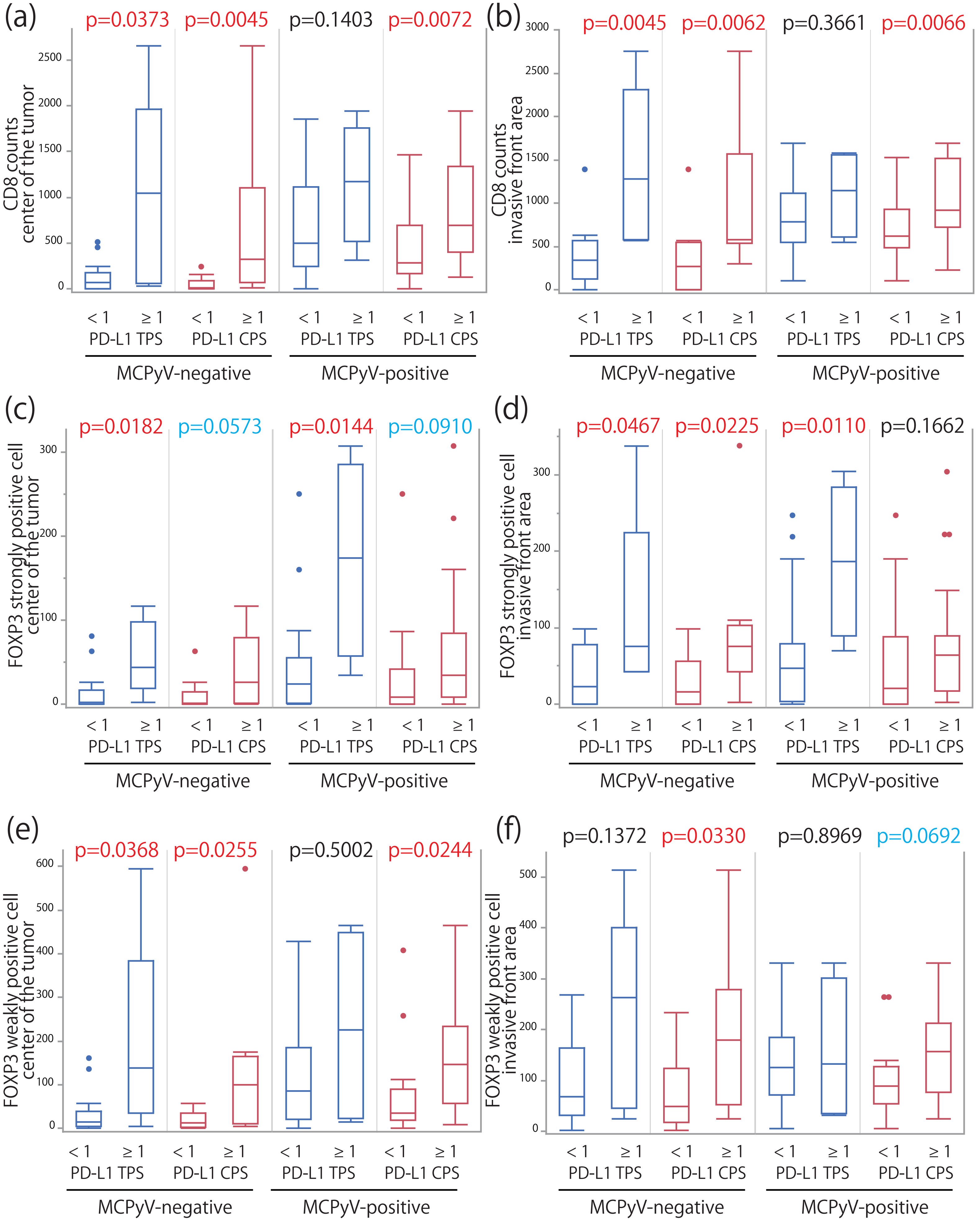

The number of CD8-positive (a, b) and high-intensity (c, d) and low-intensity (e, f) FOXP3-positive cell infiltrates was plotted separately for the tumor center (a, c, e), invasive front (b, d, f), and PD-L1 expression. Paired-sample Wilcoxon signed rank test was used to evaluate statistical significance.
