# Supplementary material for: Clinical significance of the expression of FOXP3 and TIGIT in Merkel cell carcinoma
Source: Sci Rep. 2023 Aug 12;13:13114. doi: 10.1038/s41598-023-40050-7 (PMC10423247; doi:10.1038/s41598-023-40050-7)
Supplement: Supplementary file 3 — Supplementary Information 3. [file 41598_2023_40050_MOESM3_ESM.pdf]

Supplementary Figure 2 Receiver operating characteristic (ROC) curve

(a) FOXP3
Invasive front high intensity

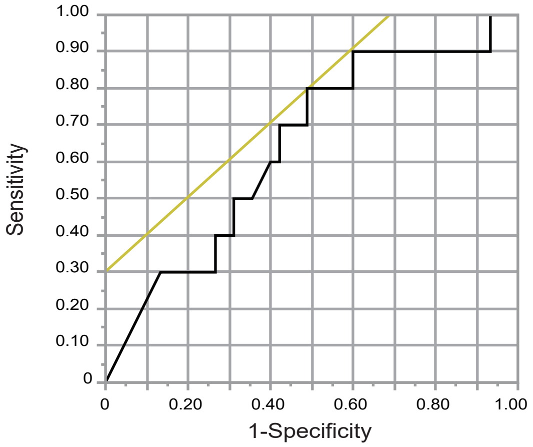

AUC: 0.64
Cut off: 48
Sensitivity-(1-Specificity): 0.31

(b) FOXP3
Invasive front low intensity

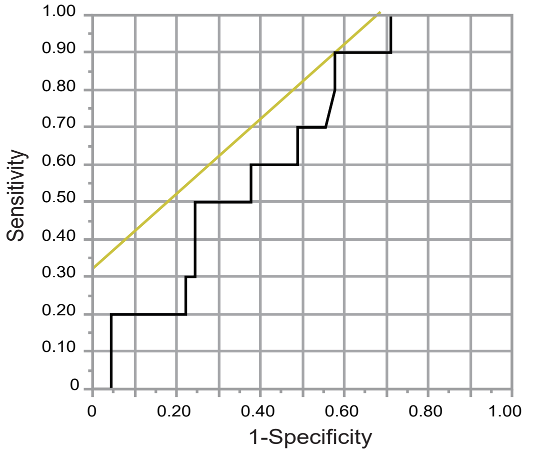

AUC: 0.64
Cut off: 128
Sensitivity-(1-Specificity): 0.32

(c) FOXP3
Center high intensity

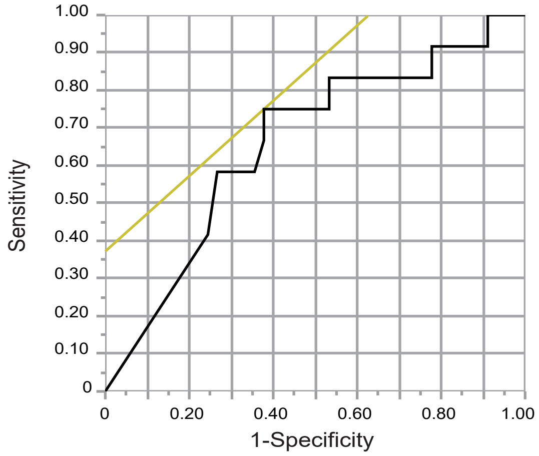

AUC: 0.66
Cut off: 11
Sensitivity-(1-Specificity): 0.37

(d) FOXP3
Center low intensity

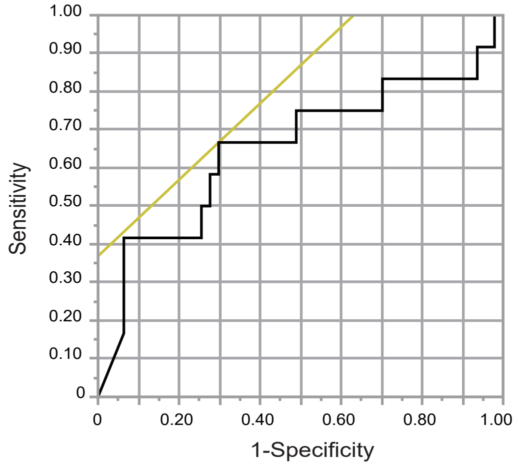

AUC: 0.66
Cut off: 58
Sensitivity-(1-Specificity): 0.32

(e) CD8 Center

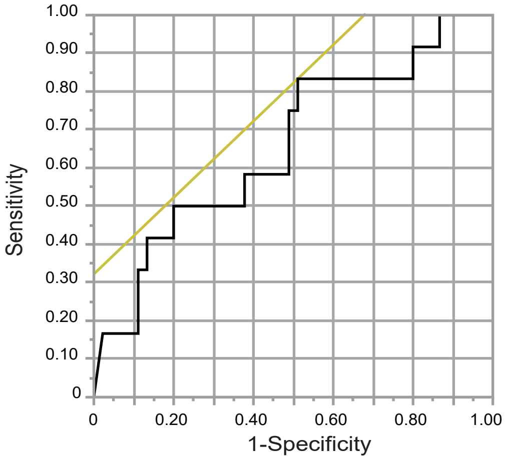

AUC: 0.65
Cut off: 186
Sensitivity-(1-Specificity): 0.37

(f) CD8 Invasive front

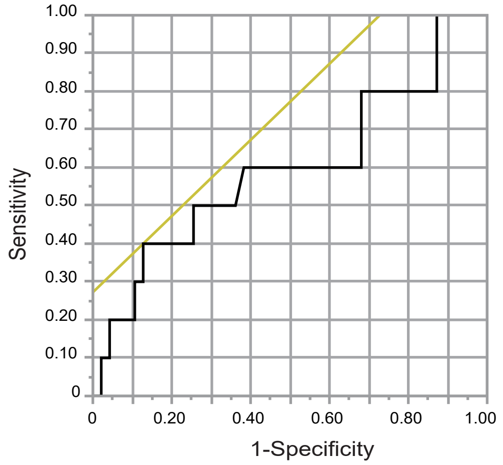

AUC: 0.60
Cut off: 276
Sensitivity-(1-Specificity): 0.27

(g) TIGIT positivity of Tumor cell

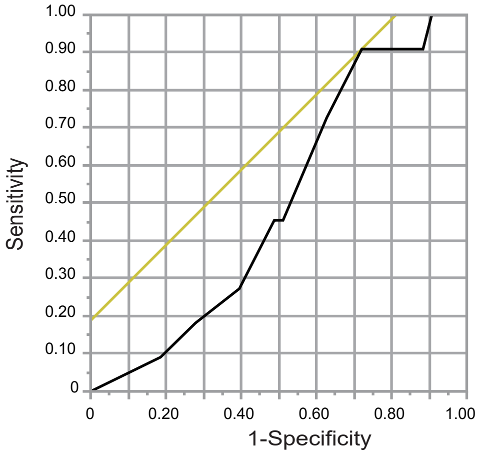

AUC: 0.50
Cut off: 30
Sensitivity-(1-Specificity): 0.19

Immunohistochemical data of FOXP3, CD8, and TIGIT cut-offs were determined by plotting ROC curves.
