# Supplementary material for: Clinical significance of the expression of FOXP3 and TIGIT in Merkel cell carcinoma
Source: Sci Rep. 2023 Aug 12;13:13114. doi: 10.1038/s41598-023-40050-7 (PMC10423247; doi:10.1038/s41598-023-40050-7)
Supplement: Supplementary file 4 — Supplementary Information 4. [file 41598_2023_40050_MOESM4_ESM.pdf]

Supplemental Figure 3 Kaplan–Meier prognostic analysis

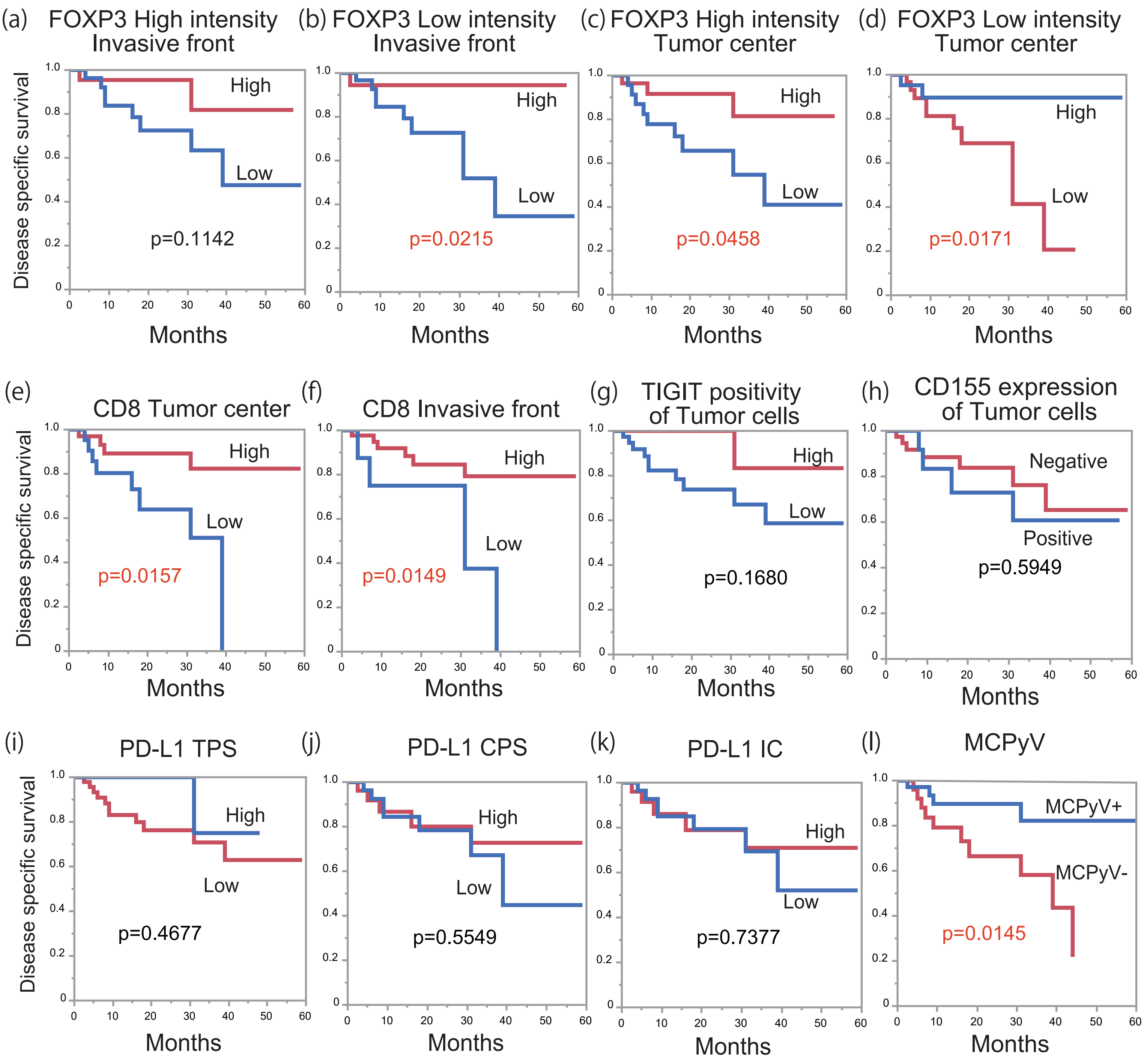

Disease-specific survival stratified by FOXP3 positivity (a to d), CD8 positivity (e, f), TIGIT expression (g), CD155 expression (h), PD-L1 expression (i to k), and MCPyV status (l)

FOXP3-positive infiltrating cells were evaluated separately for high FOXP3 expression (high-intensity) (a, c) and low FOXP3 expression (low-intensity) (b, d). CD8-positive and FOXP3-positive infiltrating cells were evaluated at the tumor center (c, d, e) and invasive front (a, b, f). Log-rank test was used to evaluate statistical significance.
